# Soft switched high gain trans inverse DC-DC converter based on three winding coupled inductor for renewable energy applications

**DOI:** 10.1038/s41598-025-92967-w

**Published:** 2025-03-17

**Authors:** Sriramkumar Venkatesan, Arun Nandagopal

**Affiliations:** School of Electrical Engineering, Vellore Institute of Technology, Vellore, 632014 Tamil Nadu India

**Keywords:** Non-isolated, Coupled inductor, Trans-inverse, Soft-switching, High gain, Passive clamp circuit, Reverse recovery issue, Energy science and technology, Engineering, Electrical and electronic engineering

## Abstract

This article discusses non-isolated, trans-inverse, coupled inductor (CI)-based, soft-switching, high-gain DC-to-DC converter topology for renewable sources. The three-winding CI is utilized to achieve a high voltage gain with a reduced turns ratio. The energy associated with the magnetic components is recycled by the passive clamp circuit through diodes, and finally it pushes the output voltage to enhance the converter voltage gain. Besides, the soft switching performance of the clamping circuit occurs during the turn-off time of the controlled switch, thereby reducing the switching loss and the reverse recovery issue on the diodes. The proposed topology benefits from a reduced component count and enhances the output voltage gain. Furthermore, the topology performance analysis is carried out using PSIM simulation, and a 250W prototype using a dSPACE controller is analyzed with theoretical expressions.

## Introduction

Electric vehicles^1^ play a major role in creating a pollution-free environment by integrating the electric vehicle charging system with any one of the renewable energy sources, making it more efficient with zero emissions. In electric vehicle charging^2^ systems integrated with renewable energy sources, the converters must manage continuous input current, handle a wide range of voltage gain with a lower duty cycle, and achieve a high voltage conversion ratio using the fewest number of components possible. This article focuses on designing the most appropriate converters to meet the above attributes.

In^3^ conventional DC-to-DC converters, such as boost and boost-derived non-isolated converters, do not achieve high gain at a large duty cycle. A larger duty cycle keeps the switch on for a long duration and increases the stress on the switch and other components, which leads to poor converter performances. The aforementioned shortcomings in conventional-type DC-to-DC converters led to the introduction of CI topology. CI used in^4^ and^5^ achieves high voltage gain while maintaining a lower duty cycle. The CI redirects its primary winding voltages to other energy storage elements, and finally, it pushes to the load side through one of the diodes. Use of CI simplifies the process of achieving high voltage gain in a DC-to-DC converter.

Voltage-boosting techniques such as the voltage multiplier (VM), switched capacitor (SC), switched inductor (SI) and cascaded technique (CT) are used by^6–8^ along with the coupled inductor. This cuts down on the number of semiconductor components, which makes the converter a little more efficient. Yet, these topologies suffer from voltage spikes in switches caused by leakage inductance, which lowers gain and efficiency. The high voltage spike causes the switch to fail. To solve the issue, use an active or passive clamp circuit across the switch. In^8^, the passive clamp circuits across the switches resolve these voltage spikes. As shown in^9–11^, the passive clamps circuits help recycle the energy from the CI to the clamping capacitor. This lowers the voltage spikes to below the output voltage and makes the converter more efficient. Even at low output voltage^12^, the hard switching and reverse recovery issues in the DC-to-DC converter negatively impact the converter’s efficiency. Therefore, to address the major problem of switching loss, the converter circuits must operate under soft-switching conditions, either zero voltage switching (ZVS) or zero current switching (ZCS). In^13^, adding soft switching along with an active or passive clamp circuit improves the ability to reduce voltage spikes and lowers the problem of reverse recovery by slowing down the current that leaks out of the leakage inductance. From a high-gain perspective, the lack of an input inductor renders this converter topology unsuitable. The problem mentioned above is fixed in^14^ by adding an input inductor to the topology. This topology works under a ZVS turn-on to achieve high voltage gain with the same turn’s ratio at high operating frequency.

Instead of using active and passive clamp circuits, the topology^15^ employs an auxiliary switch to clamp the voltage spike from the switch to the clamping capacitor. The system does not achieve a wide voltage gain. On the other hand, the topology in^16^ operates under a ZCS turn-on with a passive clamp circuit and achieves high voltage gain with the help of VM. Another topology^17^ uses the same passive clamp circuit with VM to achieve high voltage gain with fewer components. However, the topology significantly reduces the input current ripple, despite its high turn ratio. Therefore, the large size of the CI makes the topology occupy more space in the electric vehicle charging system.

Despite using two windings in the coupled inductor, the aforementioned topologies do not achieve a wide range of voltage gain, as they require more semiconductor devices and incur switching losses. Therefore, the voltage gain of the previously presented converters are limited. In order to meet the needs, the topology shown in^18^ uses a three-winding CI with a very low turns ratio. This lowers the leakage current and helps semiconductor devices with the reverse recovery problem. Moreover, a high ripple in the input inductor limits the duty cycle to 0.49. Typically, one must operate a converter at a duty ratio of 0.5 to achieve high-gain output voltage conversion. To get around the duty cycle limitation in three-winding CI^19^, adds two symmetrical VM structures in the middle of the circuit. This structure not only gets around the duty cycle limitation but also makes the voltage gain wider. The topology presented in^20^ is an extended version of topology^19^. The system combines a three-winding CI with two VMs, and the semiconductors operate under ZVS to achieve a wide range of voltage gain with a shorter duty cycle and higher efficiency. To manage the two boosting stages, the system employs an additional two power switches and two clamping circuits, resulting in a high component count.

The active switched inductor technique is used in^21^ to cut down on the number of semiconductor components and increase the voltage gain. This leads to good results in two switches with a wide voltage gain. In^22^, another topology utilizes the active switched capacitor technique with two switches, which broadens the output voltage and increases the gain through parallel charging of the switching capacitor and series discharge of the CI, leading to effective outcomes. To resolve the use of two power switches for two boosting stages, the topology^23^ employs only one switch with a new voltage lift technique by adding the capacitor in series with the first boosting stage. Hence, the other boosting stage capacitor voltage reflects the CI output voltage; as a result, the converter’s voltage gain increases with the sum of two boosting stages. Because the source and the CI connection are in series, this topology is susceptible to high input current ripple. Using passive elements to clamp the voltages of the input inductor at both ends can lower the input current ripple, as suggested by^24^. This keeps the voltage across the inductor constant and lowers the current ripple, but the topology requires an extra amount of inductors and capacitors, which makes the circuit larger and more complex.

To avoid the circuit’s complexity^25–27^, introduces the new efficient method of trans-inverse-based CI, which achieves high voltage gain with a reduced turns ratio. In this topology, the CI’s secondary winding turn ratio inversely influences the converter’s voltage gain. Despite the increased turn ratio between the primary and secondary windings, maintaining a constant voltage across the inductor and reducing the current ripple results in a narrower converter’s voltage gain.

Based on the above detailed primary requirements of a high gain DC-to-DC converter, a trans-inverse topology is proposed to achieve a wide voltage gain at a lower turn ratio by using a lower number of component counts and controlling the semiconductor devices with the soft switching technique. The key benefits of the suggested converter are

- Deliver a large voltage gain at a reduced turns ratio.
- Narrow duty cycle enables a wide voltage gain.
- Reduce the input current ripple.
- Achieve a high gain with a low component count.
- Soft switching—ZCS of the main switch.

The operating principle of the proposed topology in different modes of operation with the respective waveform is discussed in Sect. “Operating principle of proposed topology”. Section “Topology performance analysis in steady-state condition” discusses the performance analysis of the topology under steady-state conditions, considering voltage gain and stress on semiconductor devices. Section “Performance evaluation” tabulates and discusses the performance evaluation of the proposed converter in comparison with the existing topology. Section “Design specifications of the proposed converter” provides the component design specification, while Sect. “Simulation and experimental results” displays the simulation and experimental results. Section “Conclusion” presents the final summary of the findings.

## Operating principle of proposed topology

**Fig. 1:**
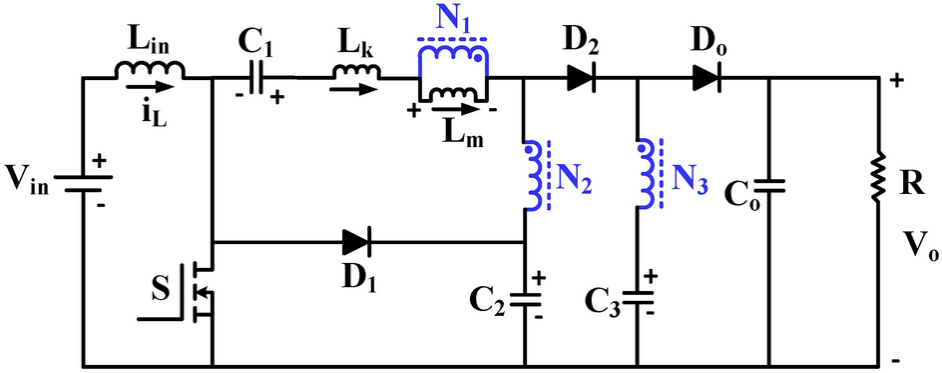
Proposed topology of trans-inverse based high gain DC-to-DC converter.

Figure 1 illustrates the proposed trans-inverse-based three-winding CI topology. The topology has two magnetic parts: a three-winding CI, which is shown by N_1_, N_2_, and N_3_; an input inductor (L_in_); a single controlled switch (S); three diodes (D_1_, D_2_, D_o_) to make sure the current flows properly; and four capacitors: three temporary voltage storage capacitors (C_1_, C_2_, C_3_) and one output load capacitor (C_o_). The primary winding consists of one leakage inductance (L_k_) and one magnetizing inductance (L_m_).

**Fig. 2:**
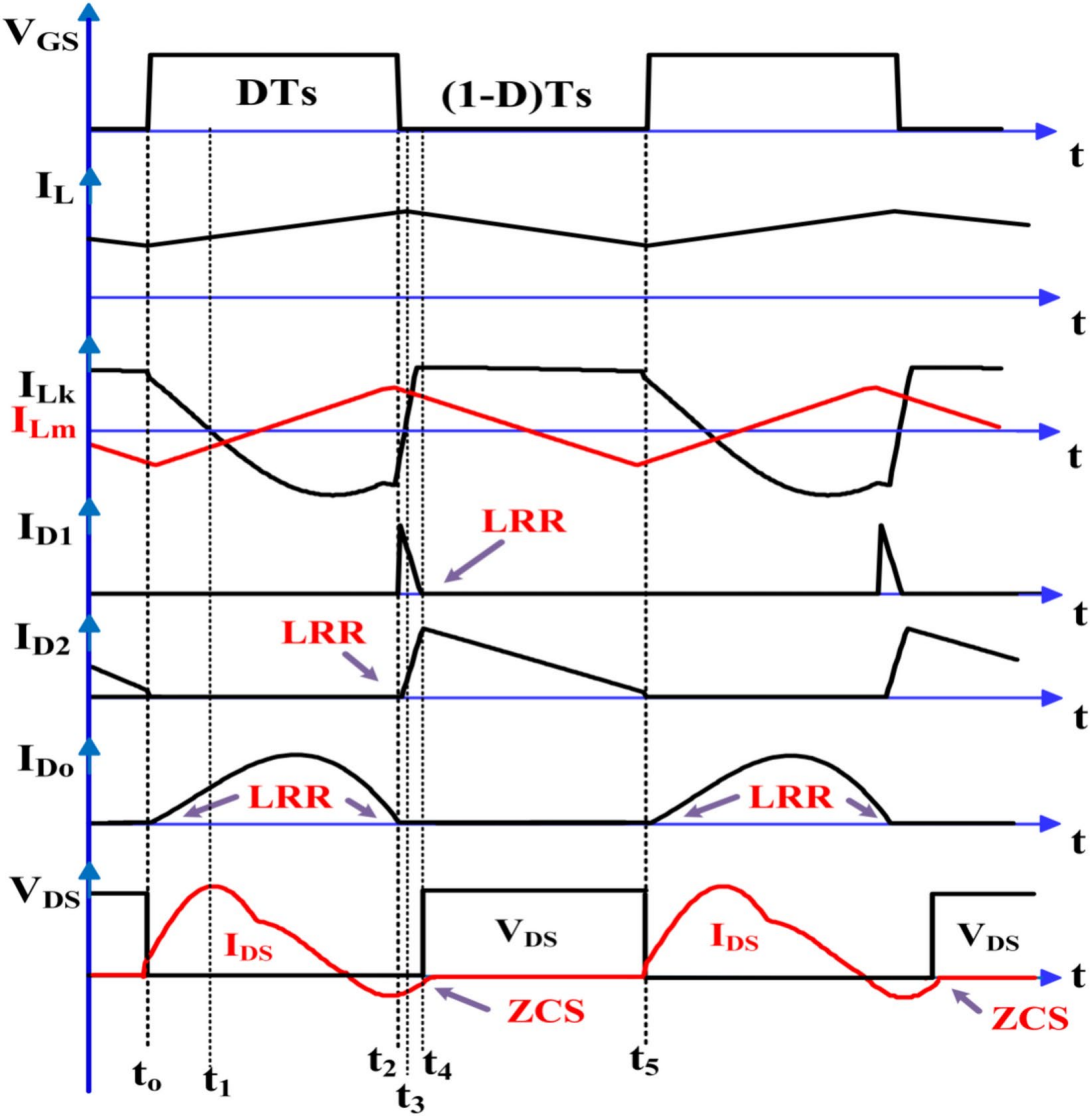
Functionality of the steady state waveforms of the proposed converter in continuous conduction mode.

The proposed converter achieves high voltage gain by setting the proper turn ratio of the secondary and tertiary windings with the help of capacitor C_3_ and diode D_2_, which enhance the boosting of the input voltage and finally drive out the boosting voltage to the output side. The formation of C_2_ and D_1_ in the clamping design relieves the maximum voltage stress across the gate-controlled switch, which has its own R_DS_-on. The series connection of the leakage inductance (L_k_) with capacitors C_1_ and C_2_ ensures the soft switching action of the proposed topology, making the tertiary winding current sinusoidal and preventing the output diode from a reverse recovery dispute. To analyze the topology with different modes of operation in continuous conduction mode, the circuit is considered ideal with no loss components. Figure 2 displays the theoretical waveform of the proposed converter, followed by the various modes of operation at the different time intervals as listed below.

**Fig. 3:**
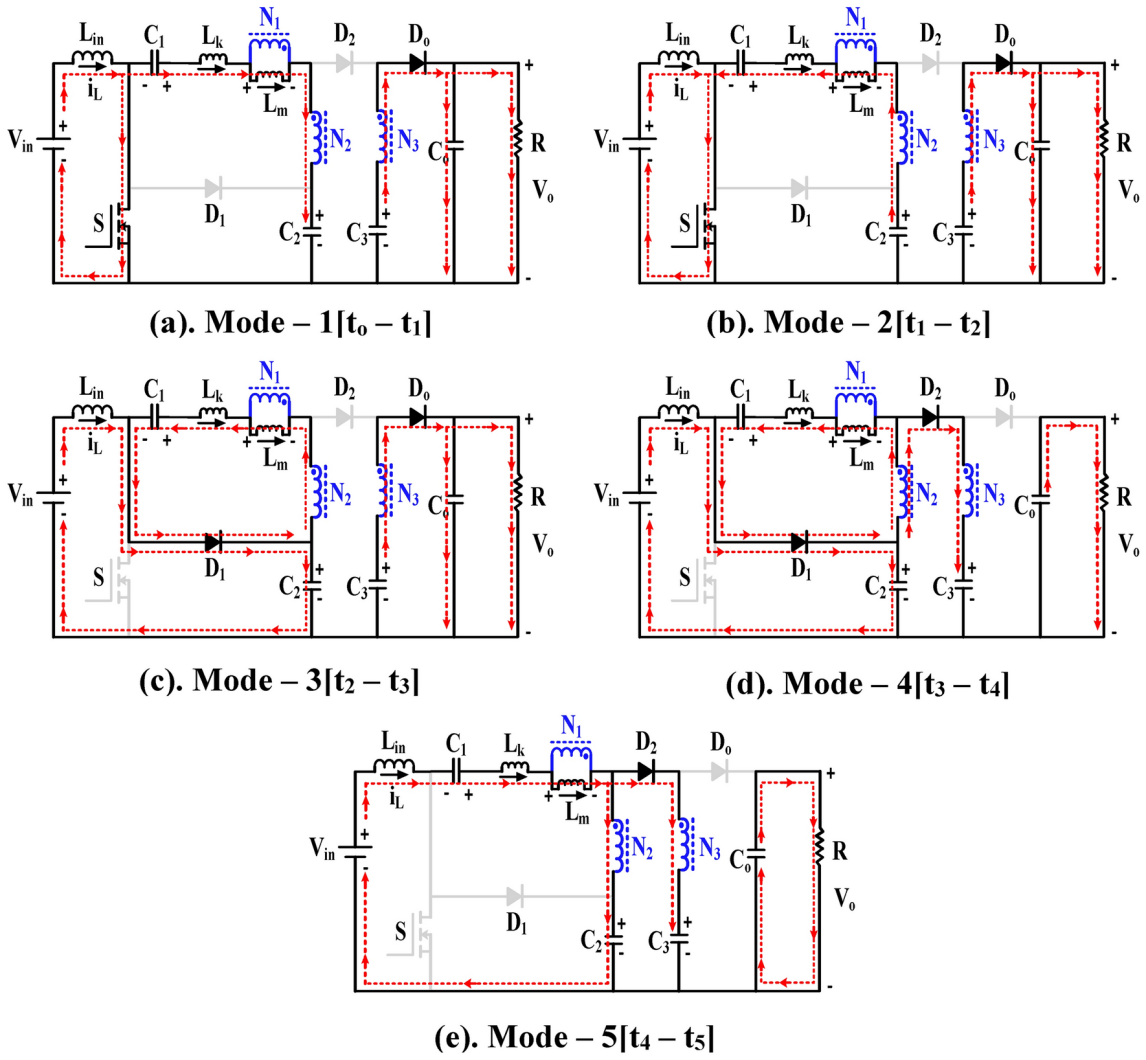
Functionality operation of the proposed converter.

Mode—1[t_o_ – t_1_], Fig. 1 shows the controlled input to the semiconductor switching device (S), which includes the turn ON and turn OFF durations. During the turn-on duration t_o_ to t_1_ as shown in Fig. 3a, the input inductor (L_in_) is energized; hence the inductor current rises linearly with a positive slope and diode D_1_ is in the OFF condition. Capacitor C_1_ and leakage inductance (L_k_) are discharged to capacitor C_2_ through primary and secondary windings. Due to the discharge of capacitor C_3_, the diode D_o_ transitions to the forward conduction state, while D_2_ transitions to the reverse blocking state, supporting both C_o_ and the load. In this short duration, the magnetizing inductor L_m_ starts rising linearly under a positive slope. This mode continues until L_k_ discharges completely.

Mode–2[t_1_–t_2_], During the interval from t_1_ to t_2_ as shown in Fig. 3b the controlled power switch remains in the ON state, and the input inductor (L_in_) continues to charge linearly. The leakage inductance (L_k_) charges in the opposite direction. The capacitor C_2_ discharges the stored energy to C_1_ and to the primary winding of CI. Throughout the operation, the capacitors C_1_, C_2_ and leakage inductance (L_k_) are connected in series to form the resonant tank and make the leakage inductance and CI current as a quasi-sine wave. But the diode D_2_ remains in the reverse blocking state due to the discharging of C_3_. At the end of this mode, the D_o_ current reaches zero, thereby achieving the Low Reverse Recovery (LRR) condition. Equations (1) to (4) represent the derived resonant frequency and output voltage during this mode.

1 $${\text{f}}_{\text{r}}=\frac{1}{2\uppi \sqrt{{\text{L}}_{\text{k}}\frac{{\text{C}}_{1}{\text{C}}_{2}}{{\text{C}}_{1}+{\text{C}}_{2}}}}$$

2 $${\text{V}}_{{\text{L}}_{\text{in}}}={\text{V}}_{\text{in}}$$

3 $${\text{V}}_{\text{Lm}}=\frac{{\text{V}}_{\text{C}1}-{\text{V}}_{\text{Lk}2}-{\text{V}}_{\text{C}2}}{{\text{n}}_{2}-1}$$

4 $$V_{o} = V_{C3} + V_{Lm} n_{3}$$

Mode–3[t_2_–t_3_], In Fig. 3c, the controlled power switch is turned OFF. Therefore, the current from the input inductor (L_in_) and leakage inductor (L_k_) flows through the clamp circuits D_1_ and C_2_, as well as the body diode of the controlled switch. This protects the power switch from the high voltage stress caused by the ZCS action. Consequently, the capacitor C_2_ starts charging. The current from the primary and secondary windings of CI reduces to zero at the end of this mode. This mode operates more quickly.

Mode–4[t_3_–t_4_], the power switch is still in the OFF state as shown in Fig. 3d. Capacitor C_1_ still charging, and the leakage inductance (L_k_) current rises to a positive slope due to the secondary winding of CI. The input inductor (L_in_) and the magnetizing inductor (L_m_) are discharging linearly. The current from the secondary winding makes the diodes D_2_ and D_o_ turn on and off respectively, while the capacitor C_3_ gets charged from the source and the load is supported by C_o_. At the end of this mode, the LRR condition turns the clamping circuit OFF as the diode D_1_ current reaches zero. The input inductor and capacitor C_3_ voltages are derived and shown in the Eqs. (5) to (7).

5 $${{\text{V}}_{\text{L}}}_{\text{in}}={\text{V}}_{\text{in}}-{\text{V}}_{\text{C}2}$$

6 $${\text{V}}_{\text{Lm}}=\frac{{\text{V}}_{\text{C}1}-{\text{V}}_{\text{Lk}4}}{{\text{n}}_{2}-1}$$

7 $${\text{V}}_{\text{C}3}={\text{V}}_{\text{C}2}+{\text{V}}_{\text{Lm}}({\text{n}}_{2}-{\text{n}}_{3})$$

Mode–5[t_4_–t_5_], as the power switch is still in the OFF state, the previous state of leakage inductance (L_k_) current remains unchanged. The capacitor C_1_ is discharging to C_2_ through the secondary winding of CI. While the input inductor L_in_ is continuously discharging, the source continues to charge the capacitor C_3_ through D_2_ and the tertiary winding of CI. The capacitor C_o_ supports the load and keeps the diode D_o_ in a reverse blocking state as shown in Fig. 3e. The power switch turns ON at the end of this mode.

## Topology performance analysis in steady-state condition

### Voltage step-up conversion

Apply the volt-second balance to the inductor during the switch ON and switch OFF instances, taking into account modes 2 and 4, to calculate the gain of the proposed converter. Using Eqs. 2, 3, 4, 5, 6, the volt-second balance relations are given below.

8 $$\frac{{\text{V}}_{\text{C}1}-{\text{V}}_{\text{Lk}2}-{\text{V}}_{\text{C}2}}{{\text{n}}_{2}-1}\text{D}+\frac{{\text{V}}_{\text{C}1}-{\text{V}}_{\text{Lk}4}}{{\text{n}}_{2}-1}\left(1-\text{D}\right)=0$$

9 $${\text{V}}_{\text{in}}\text{D}+\left({\text{V}}_{\text{in}}-{\text{V}}_{\text{C}2}\right)\left(1-\text{D}\right)=0$$

Based on equation the Eqs. (8) and (9), the following capacitor voltages are obtained,

10 $${\text{V}}_{\text{C}1}=\text{D}({\text{V}}_{\text{Lk}2}+{\text{V}}_{\text{C}2})+{\text{V}}_{\text{Lk}4}(1-\text{D})$$

11 $${\text{V}}_{\text{C}2}=\frac{{\text{V}}_{\text{in}}}{1-\text{D}}$$

By substituting the value of V_C2_ in V_C1_ (Eqs. (10) and (11)), the equation for V_C1_ is expressed in (12)

12 $${\text{V}}_{\text{C}1}=\text{D}({\text{V}}_{\text{Lk}2}+\frac{{\text{V}}_{\text{in}}}{1-\text{D}})+{\text{V}}_{\text{Lk}4}(1-\text{D})$$

From Eqs. (6) and (7), the value of V_C3_ is expressed in Eq. (13)

13 $${\text{V}}_{\text{C}3}={\text{V}}_{\text{C}2}+\frac{{\text{V}}_{\text{C}1}-{\text{V}}_{\text{Lk}4}}{{\text{n}}_{2}-1}({\text{n}}_{2}-{\text{n}}_{3})$$

Equation (14) projects the relationship between the input and output voltage gain of the proposed converter by substituting the values of V_C1_, V_C2_, and V_C3_ in V_O_.

14 $${\text{V}}_{\text{o}}=\frac{{\text{n}}_{2}-1+{\text{Dn}}_{2}+{\text{n}}_{3}}{({\text{n}}_{2}-1)(1-\text{D})}{\text{V}}_{\text{in}}+\frac{{\text{n}}_{2}-{\text{n}}_{3}}{{\text{n}}_{2}-1}({\text{V}}_{\text{Lk}2}-{\text{V}}_{\text{Lk}4})$$

The differences in leakage inductance current, as shown in Fig. 3a,b, negatively impact the output voltage gain in the above output equation. Therefore, the voltage gain of the proposed converter is simplified to Eq. (15).

15 $$\frac{{\text{V}}_{\text{o}}}{{\text{V}}_{\text{in}}}=\frac{{\text{n}}_{2}\left(1+\text{D}\right)+{\text{n}}_{3}-1}{({\text{n}}_{2}-1)(1-\text{D})}$$

**Fig. 4:**
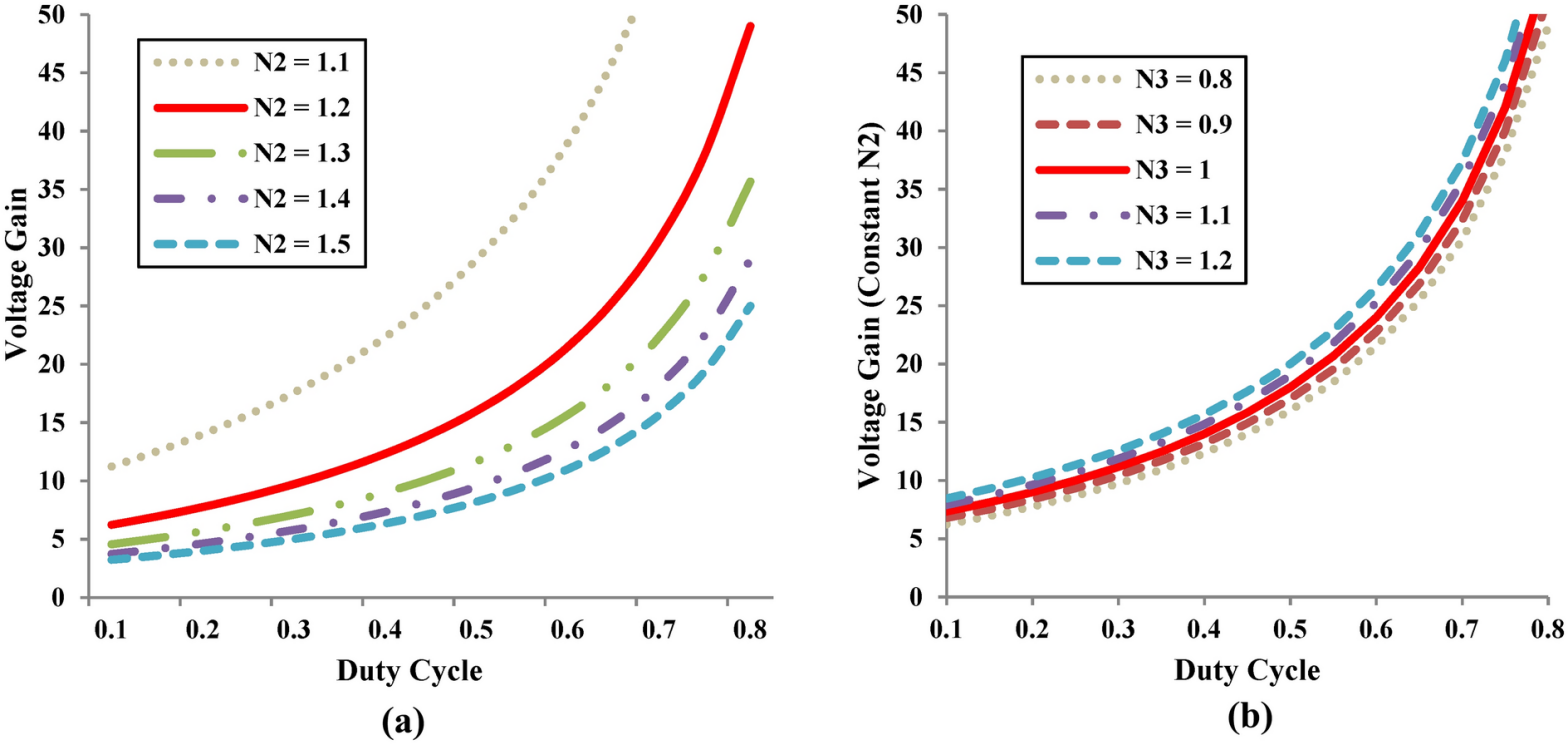
Variation of voltage gain with respect to control input (D). (**a**) For different cases of N_2_ turns at constant N_3_. (**b**) For different cases of N_3_ turns at constant N_2_.

The voltage gain of the proposed converter depends on duty cycle and CI’s secondary and tertiary winding turn ratios. Figure 4a depicts the variation of proposed topology voltage gain with respect to the variation of the control input at constant n_3_ = 0.8 for different cases of n_2_ (1.1, 1.2, 1.3, 1.4, 1.5). Similar figure is portrayed in Fig. 4b for different cases of n_3_ (0.8, 0.9, 1, 1.1, 1.2) at constant n_2_ = 1.2.

**Fig. 5:**
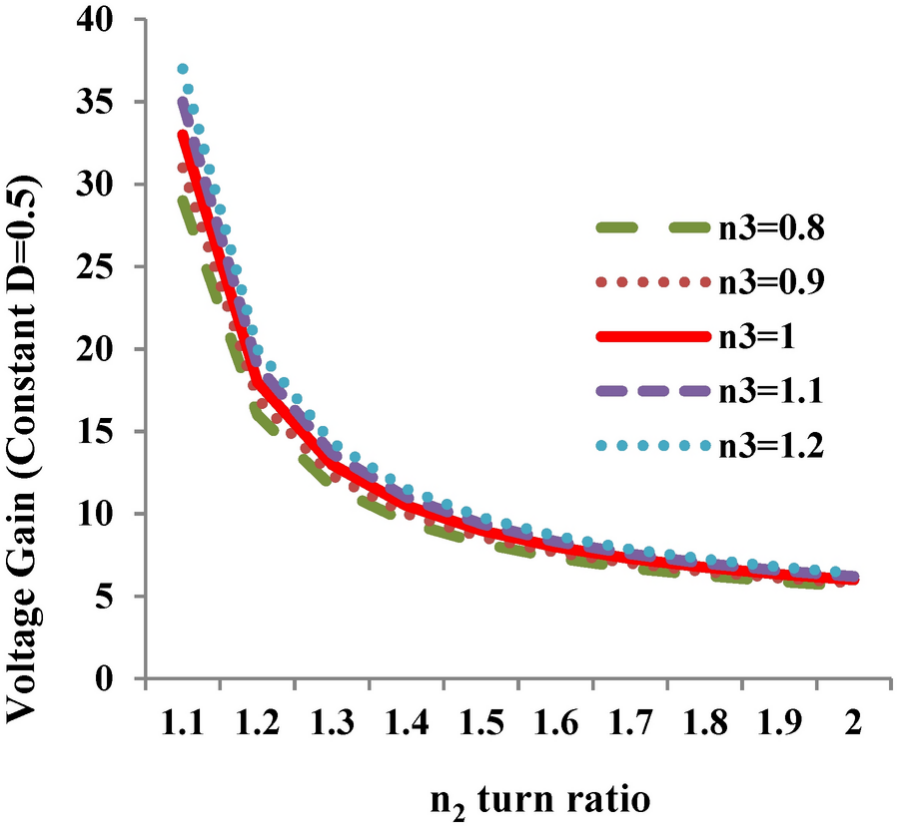
Variation of voltage gain with respect to n_2_ turns ratio for different cases of n_3_.

**Fig. 6:**
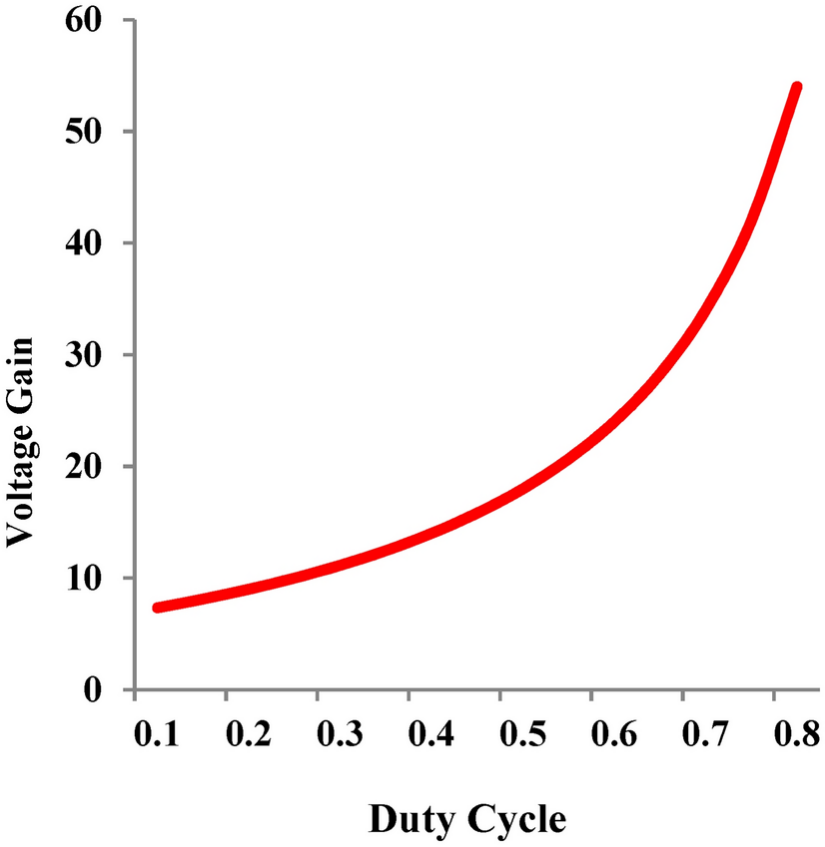
Variation of voltage gain when N_2_ = 1.2 and N_3_ = 1.

It is inferred from Fig. 4a, that the voltage gain varies when the turns ratio of n_2_ is selected between 1.1 to 1.5 and it is observed that the voltage gain is maximum for 1.1 ≤ n_2_ ≤ 1.2. Hence as the turns ratio of n_2_ decreases, the voltage gain reaches maximum value and therefore the suggested topology exhibits a trans-inverse characteristics. Extending the similar analysis to Fig. 4b, the turns ratio of n_3_ value is selected between 0.8 ≤ n_3_ ≤ 1. Based on the above range of values of n_2_ and n_3_ voltage gain of Eq. (15) is displayed in Fig. 5 at D = 0.5. Due to the trans-inverse characteristics, n_2_ is varied between 1.1 and 1.2 for the different cases of turns ratio of n_3_ (case 1: n_3_ = 0.8; case 2: n_3_ = 0.9 and case 3: n_3_ = 1). Consequently, the voltage gain of the suggested topology shown in Fig. 6 is achieved by varying the controlled input D by fixing the value of n_2_ = 1.2 and n_3_ = 1.

### Stress on semiconductor devices

The maximum voltage stress across the switch, diodes D_1_, D_2_ and D_o_ during one switching cycle is written as follows,

16 $${\text{V}}_{\text{sw}}=\frac{{\text{V}}_{\text{in}}}{1-\text{D}}=\frac{{\text{n}}_{2}-1}{{\text{n}}_{2}\left(1+\text{D}\right)+{\text{n}}_{3}-1}{\text{V}}_{\text{o}}$$

17 $${\text{V}}_{\text{sw}}={\text{V}}_{\text{D}1}=\frac{{\text{n}}_{2}-1}{{\text{n}}_{2}\left(1+\text{D}\right)+{\text{n}}_{3}-1}{\text{V}}_{\text{o}}$$

18 $${\text{V}}_{\text{D}2}=\frac{{\text{n}}_{2}(1+\text{D})}{{\text{n}}_{2}\left(1+\text{D}\right)+{\text{n}}_{3}-1}{\text{V}}_{\text{o}}$$

19 $${\text{V}}_{\text{Do}}=\frac{{\text{n}}_{2}}{{\text{n}}_{2}\left(1+\text{D}\right)+{\text{n}}_{3}-1}{\text{V}}_{\text{o}}$$

Using the Eqs. (16) to (19), the calculated diode voltages are very less compared to the output voltage of the converter and it proves that the passive clamping circuit of the switch works efficiently for the proposed converter.

**Fig. 7:**
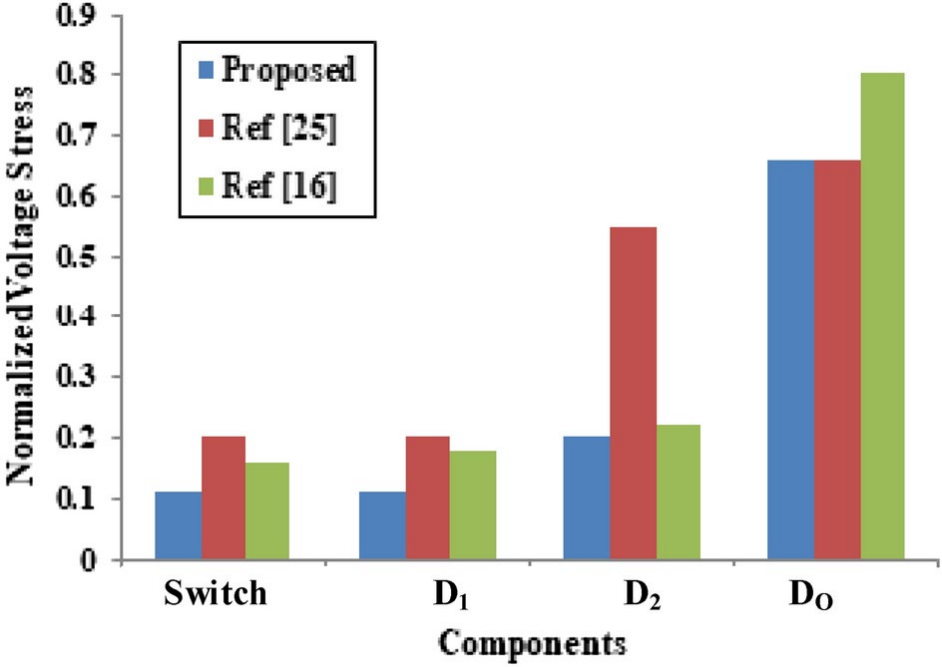
Normalized voltage stress of the semiconductors.

By considering the output voltage as the base value, the normalized voltage stresses of the semiconductors are displayed in Fig. 7.

It is evident from Fig. 7 that the voltage stress of the switch and diodes are lower than the semiconductor devices reported in^25^ and^16^.

**Fig. 8:**
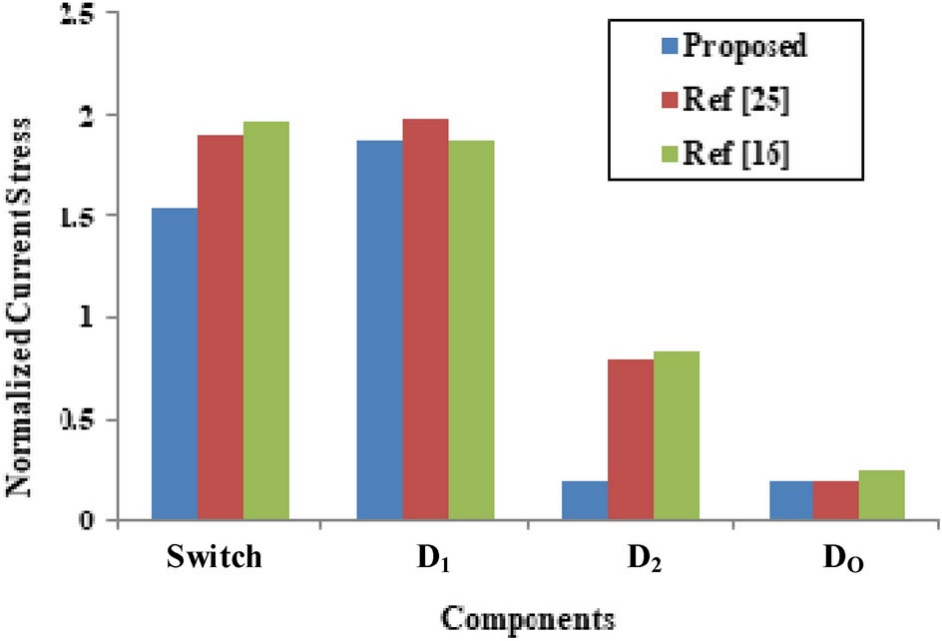
Normalized current stress of the semiconductors.

The typical calculated value of average current in inductor and diodes are given in Eqs. (20) to (22).

20 $${\text{i}}_{{{\text{L}}_{\text{in}}}_{\text{avg}}}=\frac{{\text{n}}_{2}\left(1+\text{D}\right)+{\text{n}}_{3}-1}{({\text{n}}_{2}-1)(1-\text{D})}{\text{V}}_{\text{o}} \frac{1}{\text{R}}$$

21 $${\text{i}}_{{\text{Lm}}_{\text{avg}}}=\left({\text{n}}_{2}-1\right){\text{V}}_{\text{o}} \frac{1}{\text{R}}$$

22 $${\text{i}}_{{\text{D}1}_{\text{avg}}}={\text{i}}_{{\text{D}2}_{\text{avg}}}={\text{i}}_{{\text{Do}}_{\text{avg}}}\ge {\text{V}}_{\text{o}} \frac{1}{\text{R}}$$

where, R is the load resistance of the converter. The normalized current stress of the devices are displayed in Fig. 8 by considering the input current as the base value. The normalized switch current shown in Fig. 8 is 1.5 p.u whereas, the normalized switch current reported in^25^ and^16^ is higher than 1.5 p.u and it is equal to 1.9 and 1.97 p.u respectively. Due to this, the corresponding diode D_1_ across the switch draws current around 1.8 p.u and it is lower than the diode currents reported in^25^ and^16^. The normalized output diode current of D_o_ is very low whereas the diode D_2_ normalized current is equal to 1.8 p.u as it is connected between the secondary and tertiary winding of the coupled inductor. Hence the proposed topology normalized switch/diode currents are lower than the device currents reported in^25^ and^16^.

## Performance evaluation

**Fig. 9:**
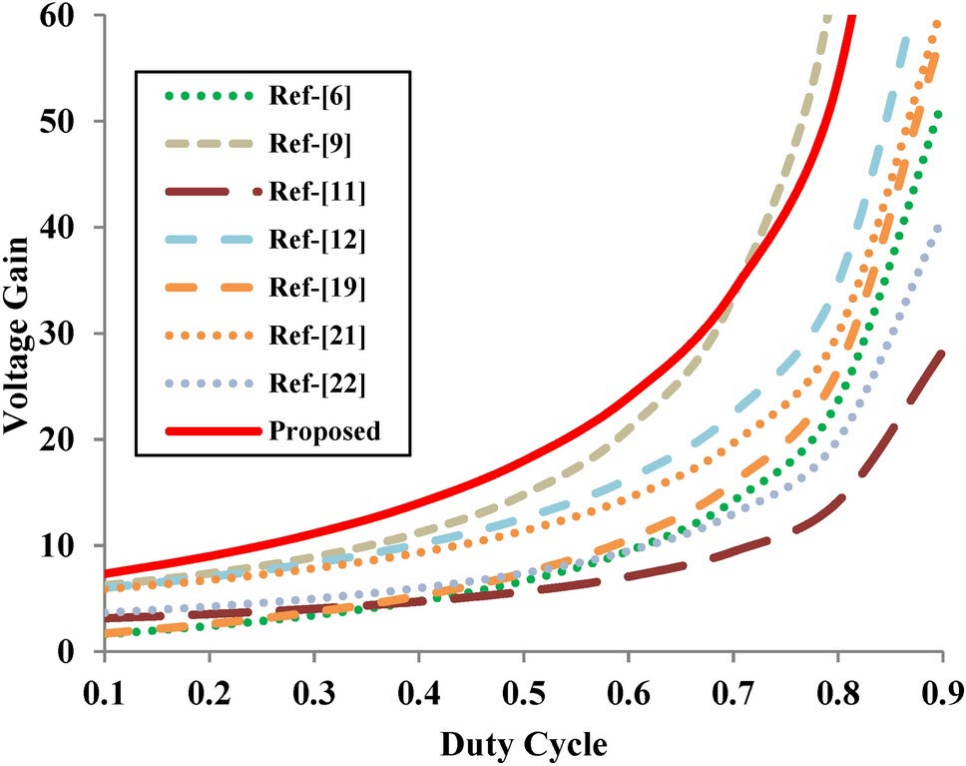
Voltage gain comparison of proposed converter with existing topologies.

The advantages of the suggested converter are examined by comparing with the most recent converters as projected in Table 1. In order to compare the performance of the different converters, Fig. 9 shows the voltage gain with the parameters n_2_ = 1.2 and n_3_ = 1 for converters with three-winding CI topologies and n = n_2_ + n_3_ = 2.2 for converters with two-winding CI. In addition to having fewer components, it is clear that the suggested converter has a high voltage gain and lower voltage stress across the MOSFET for a control input of D = 0.5. As a result, conduction losses and cost can be greatly decreased by using low voltage rating MOSFETs with lower R_DS_-on values.

**Table 1:** Performance evaluation of proposed converter with existing topologies.

Topology in	Component count		Voltage gain	Switch voltage stress	Diode voltage stress	Soft switching	Wide gain		
	CI winding	Total					D = 0.4	D = 0.5	D = 0.6
^6^	1^2w^	10	$\frac{3\text{nD}+\text{n}-\text{D}-1}{\text{n}+\text{D}-1-\text{nD}}$	$\frac{2\text{n}-2}{3\text{nD}+\text{n}-\text{D}-1}$	$\frac{4\text{n}-2}{3\text{nD}+\text{n}-\text{D}-1}$	No	4.8	6.7	9.5
^9^	1^2w^	12	$\frac{1+\text{D}+2\text{n}(1-\text{D})}{{(1-\text{D})}^{2}}$	$\frac{(1-\text{D}){\text{V}}_{\text{o}}}{1+\text{D}+2\text{n}(1-\text{D})}$	$\frac{2{\text{nV}}_{\text{o}}}{1+\text{D}+2\text{n}(1-\text{D})}$	No	`11.2	14.8	21
^11^	1^2w^	10	$\frac{2\text{n}-1}{\left(\text{n}-1\right)\left(1-\text{D}\right)}$	$\frac{\text{n}-1}{2\text{n}-1}$	$\frac{\text{n}}{2\text{n}-1}$	No	4.7	5.7	7.1
^12^	1^2w^	12	$\frac{3+\text{n}(1+\text{D})}{\left(1-\text{D}\right)}$	$\frac{{\text{V}}_{\text{o}}}{3+\text{n}(1+\text{D})}$	$\frac{(\text{n}+1){\text{V}}_{\text{o}}}{3+\text{n}(1+\text{D})}$	No	10.1	12.6	16.3
^19^	1^3w^	12	$\frac{1+2\text{nD}+\text{D}}{1-\text{D}}$	$\frac{{\text{V}}_{\text{o}}}{1+2\text{nD}+\text{D}}$	$\frac{{\text{nV}}_{\text{o}}}{1+2\text{nD}+\text{D}}$	Yes	5.3	7.4	10.6
^21^	1^3w^	9	$\frac{3+\text{n}+\text{D}}{1-\text{D}}$	$\frac{{\text{V}}_{\text{o}}}{3+\text{n}+\text{D}}$	$\frac{{\text{nV}}_{\text{o}}}{3+\text{n}+\text{D}}$	Yes	9.3	11.4	14.5
^22^	1^3w^	10	$\frac{{\text{Dn}}_{3}+{\text{n}}_{2}+2}{1-\text{D}}$	$\frac{{\text{V}}_{\text{g}}}{1-\text{D}}$	$\frac{{DV_{g} n_{3} }}{1 - D}$	Yes	6.0	7.4	9.5
^23^	1^3w^	12	$\frac{\text{D}{(\text{n}}_{2}-1)-{\text{n}}_{2}+2}{(1-{\text{D})}^{2}(1-{\text{n}}_{3})}$	$\frac{\left(1-\text{D}\right)\left(1-{\text{n}}_{3}\right){\text{V}}_{\text{o}}}{\text{D}{(\text{n}}_{2}-1)-{\text{n}}_{2}+2}$	$\frac{\left(1-{\text{n}}_{3}\right){\text{nV}}_{\text{o}}}{\text{D}{(\text{n}}_{2}-1)-{\text{n}}_{2}+2}$	Yes	–	–	–
^25^	1^3w^	12	$\frac{2+{\text{n}}_{2}(2-\text{D})-{\text{n}}_{3}(1+\text{D})}{(1-\text{D})(1-{\text{n}}_{3})}$	$\frac{(1-{\text{n}}_{3}){\text{V}}_{\text{o}}}{2+{\text{n}}_{2}(2-\text{D})-{\text{n}}_{3}(1+\text{D})}$	$\frac{(1+{\text{n}}_{2}-{\text{n}}_{3}){\text{V}}_{\text{o}}}{2+{\text{n}}_{2}(2-\text{D})-{\text{n}}_{3}(1+\text{D})}$	Yes	–	–	–
Proposed	1^3w^	10	$\frac{{\text{n}}_{2}\left(1+\text{D}\right)+{\text{n}}_{3}-1}{\left({\text{n}}_{2}-1\right)\left(1-\text{D}\right)}$	$\frac{{(\text{n}}_{2}-1){\text{V}}_{\text{o}}}{{\text{n}}_{2}\left(1+\text{D}\right)+{\text{n}}_{3}-1}$	$\frac{{\text{n}}_{2}{\text{V}}_{\text{o}}}{{\text{n}}_{2}\left(1+\text{D}\right)+{\text{n}}_{3}-1}$	Yes	14	18	24

*2w and *3w refers to two-winding and three-winding respectively.

**Fig. 10:**
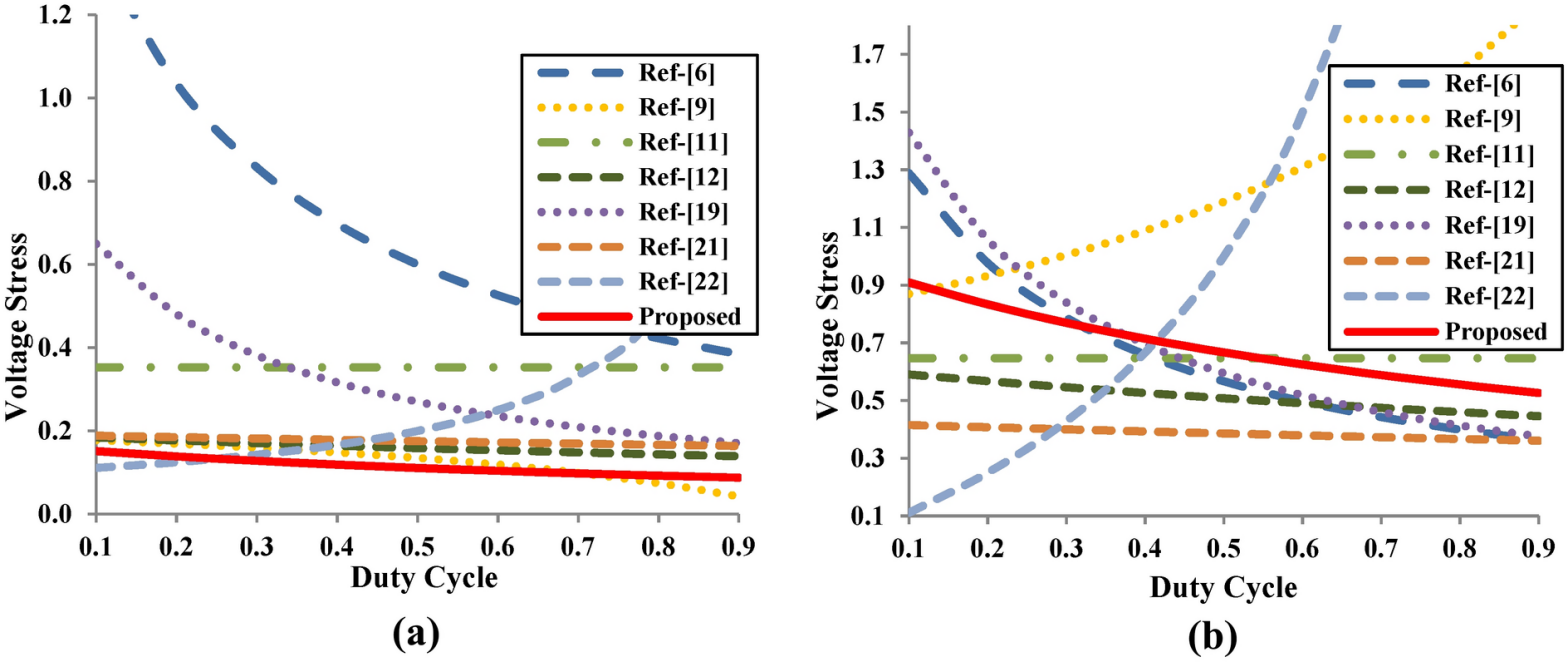
Voltage stress comparison of proposed converter with existing topologies. (**a**) Voltage stress of switch (**b**) Voltage stress of diode.

Figure 10a,b illustrate the voltage stress on the switch (S) and output diode (D_o_) for all topologies by taking the value of D = 0.5. The clamping action of C_2_ and D_1_ in the proposed converter effectively reduces the voltage stress on the switch. The voltage stress on the output diode is not beyond the output voltage of the converter. The wider voltage gain in a lower duty ratio is also one of the unique features of the proposed converter.

To illustrate the performance of the non-ideal mode of the converter, the non-ideal gain of the proposed converter is derived^26^ and shown below in Eq. (23).

23 $$\frac{{V_{o} }}{{V_{in} }} = \left( {\frac{{\left( {n_{2} \left( {1 + D} \right) + n_{3} - 1} \right) - \frac{{D\prime \left( {V_{FD1} + V_{FD2} } \right) + DV_{FDo} }}{{V_{in} }}}}{{\left( {n_{2} - 1} \right)\left( {1 - D} \right)}}X\frac{R}{{R + R_{eq} }}} \right)$$

where $${\text{R}}_{\text{eq}}={\text{R}}_{\text{L}}+{\text{DR}}_{{\text{Ds}}_{(\text{on})}}+{\text{R}}_{\text{c}1}+{\text{R}}_{\text{c}2}+{\text{R}}_{\text{c}3}+{\text{R}}_{\text{co}}+{\text{R}}_{\text{Lk}}+{\text{R}}_{\text{n}1}+{\text{R}}_{\text{n}2}+{\text{R}}_{\text{n}3}$$

And the parasitic parameters of the components/devices are given below.

(R_L_=15mΩ, R_DS(on)_=5.6mΩ, R_C1_=5mΩ, R_C2_=15mΩ, R_C3_=10mΩ, R_Co_=75mΩ, R_Lk_=5mΩ, R_n1_=12mΩ, R_n2_=18mΩ, R_n3_=12mΩ, V_FD1_=V_FD2_=V_FDo_=0.8V)

**Fig. 11:**
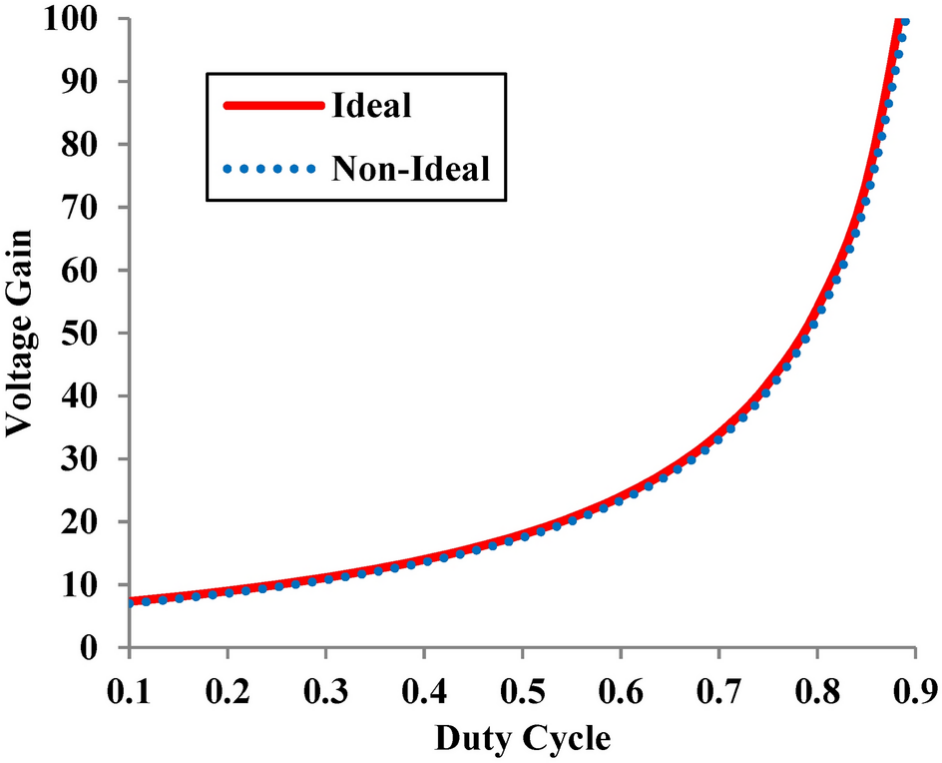
Voltage gain of the proposed converter in ideal and non-ideal mode.

In Eq. (23), R can be approximated to R + R_eq_, because R is very high. Hence the effect of parasitic elements are negligible and the non-ideal voltage gain coincides with the ideal voltage gain as observed in Fig. 11.

**Fig. 12:**
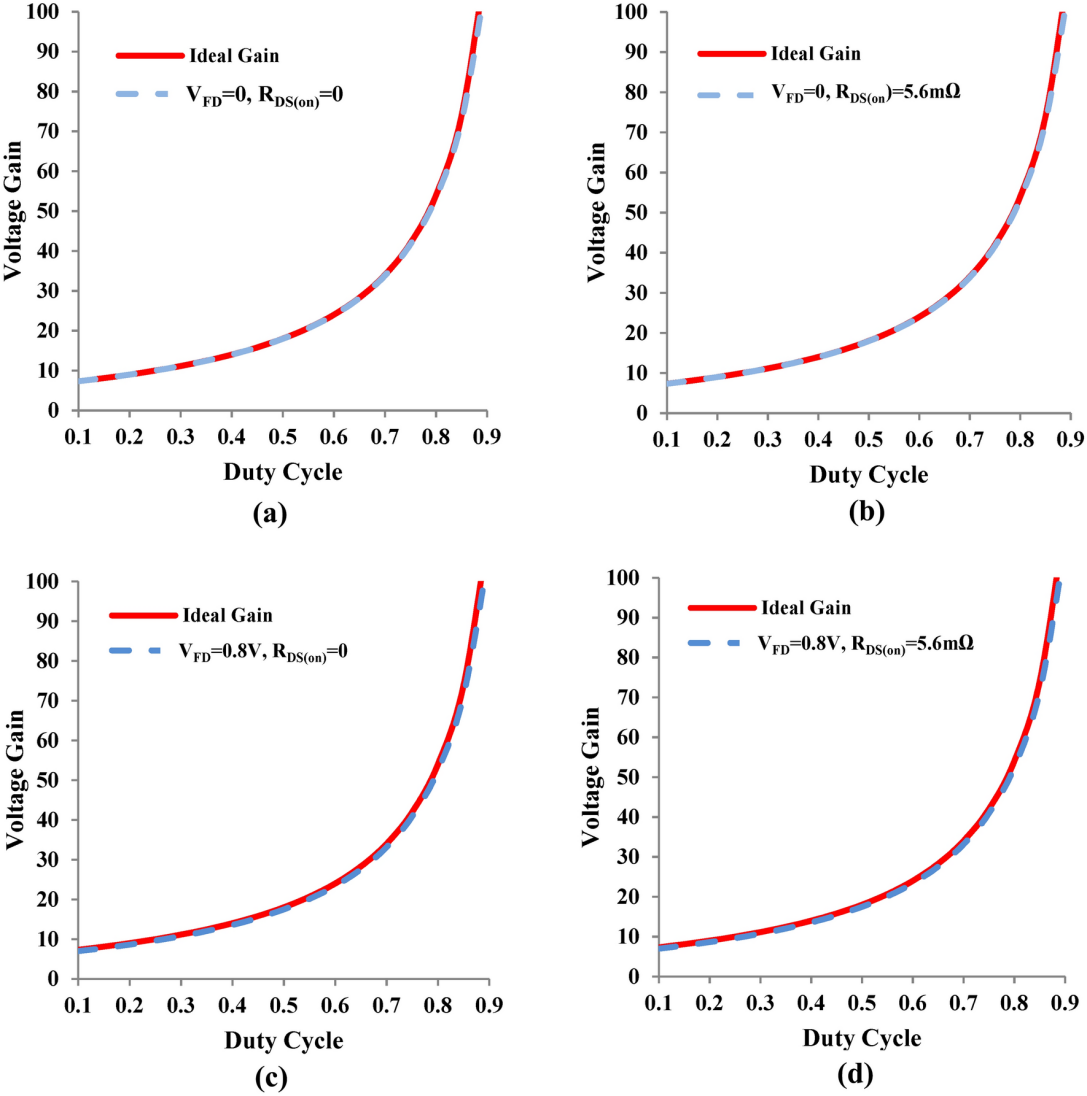
Sensitivity analysis of non-ideal voltage gain of the proposed converter with respect to device voltage drops (V_FD_ and R_DS(on)_).

The sensitivity of the non-ideal voltage gain is tested with respect to forward voltage drop of diodes and R_DS(on)_ of the controlled switch. The effect of variation of R_DS(on)_ is negligible as it can be inferred from the Fig. 12a,b, whereas the effect of the forward voltage drop of the diodes is observed in Fig. 12c,d, but it is not dominant. Consequently the proposed converter is insensitive to parasitic variations.

## Design specifications of the proposed converter

Table 2 presents, the design of the components. For D = 0.5, the voltage stress on the main switch/diode is significantly decreased, and the input inductor is designed with the value of D = 0.5 to handle the low input current ripple of 15%. Hence, the Eq. (24) is derived and projects the change in the ripple current.

24 $$\Delta i_{L} = \frac{{V_{in} }}{{L_{in} f_{s} }}D$$

**Table 2:** Components specification of the proposed topology.

Components	Values
Output power	250W
Input voltage	25 V
Output voltage	450 V
Switching frequency	50 kHz
Input/magnetizing inductor	180μH/220μH (EE42/21/20)
Turn ratio of CI (n_1_:n_2_:n_3_)	1:1.2:1 (EE42/21/20)
Capacitors (C_1_,C_2_,C_3_,C_o_)	5.6μF, 20μF, 15μF, 100μF
Switch (S)	IPP076N15N5
Diodes (D_1_,D_2_,D_o_)	MUR820/2560

The current ripple of the magnetizing inductance is calculated by using the expression (25) based on a 10% current ripple.

25 $$\Delta i_{Lm} = \frac{{V_{Lm} }}{{L_{m} f_{s} }}D$$

The maximum output voltage ripple on the load side is derived based on a 2% voltage ripple, and the expression of the output filter capacitor is shown in Eq. (26).

26 $$\Delta V_{Co} = \frac{{I_{o} }}{{C_{o} f_{s} }}D$$

The remaining capacitors of the proposed topology (C_1_, C_2_, C_3_) are designed according to modes of operation as portrayed in Fig. 3, for achieving high voltage gain and presented in Eqs. (27) to (29).

27 $$\Delta V_{C2} = \frac{{I_{in} }}{{C_{1} f_{s} }}\left( {1 - D} \right)$$

28 $$\Delta V_{C2} = \frac{{I_{in} }}{{C_{2} f_{s} }}\left( {1 - D} \right)$$

29 $$\Delta V_{C3} = \frac{{I_{in} }}{{C_{3} f_{s} }}\left( {1 - D} \right)$$

## Simulation and experimental results

The functionality of the proposed high gain trans-inverse topology is tested in PSIM with the following parameters: V_in_ = 25 V, f_s_ = 50 kHz, D = 0.5 for the output power of 250W, output voltage = 450 V and load current = 0.55A. The magnetizing components, capacitive components and the semiconductor switching devices are indexed in Table 2.

**Fig. 13:**
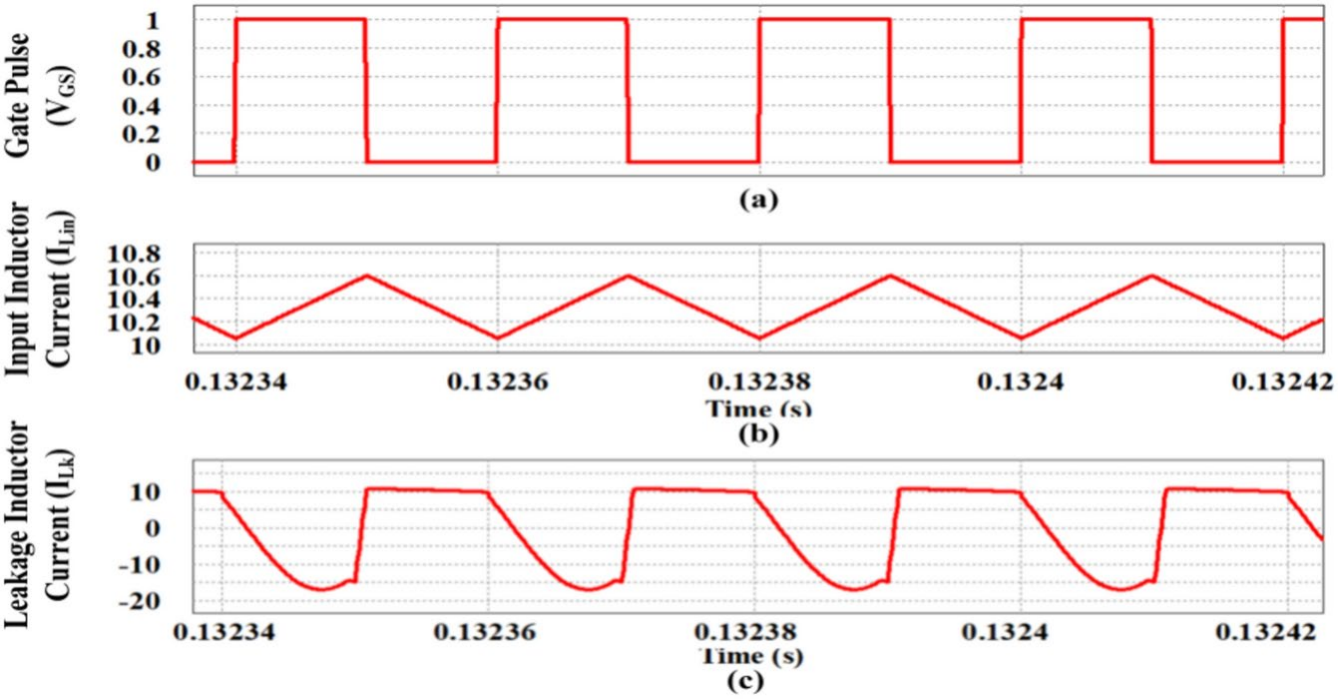
Simulation of inductor current waveforms. (**a**) Gate pulse (V_GS_), (**b**) Input inductor current (I_Lin_), (**c**) Leakage inductance current (I_Lk_).

The controlled input (V_GS_) of the switching device (S) with equal ON time and OFF time is shown in Fig. 13a. As per the Fig. 3, the inductor current with a ripple value of 0.6A is shown in Fig. 13b along with the leakage inductance current in Fig. 13c.

**Fig. 14:**
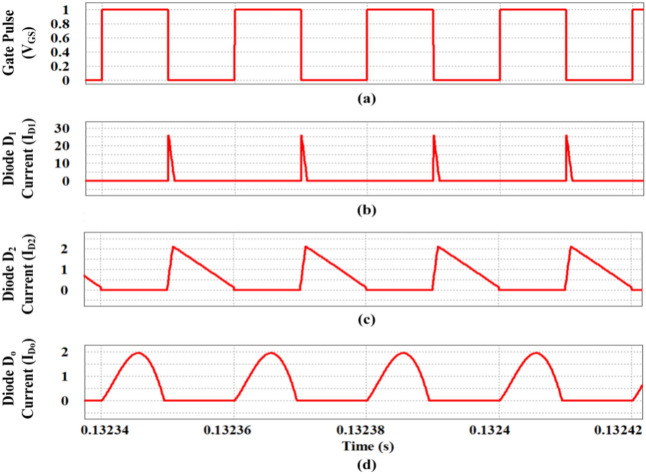
Simulation of diode currents waveforms (**a**) Gate pulse (V_GS_), (**b**) Diode D_1_ current (I_D1_), (**c**) Diode D_2_ current (I_D2_), (**d**) Diode D_o_ current (I_Do_).

During the turn OFF condition of (S) shown in Fig. 14a, diode D_1_ conducts for a short duration with peak value of 25A as projected in Fig. 14b. The remaining diode currents (I_D2_ and I_D3_) are portrayed in Fig. 14c,d and it is interpreted that diode D_o_ conducts during turn ON time of (S) while the diode D_2_ conducts during the turn OFF time. The magnitude of I_Do_ and I_D2_ are less than 2A.

**Fig. 15:**
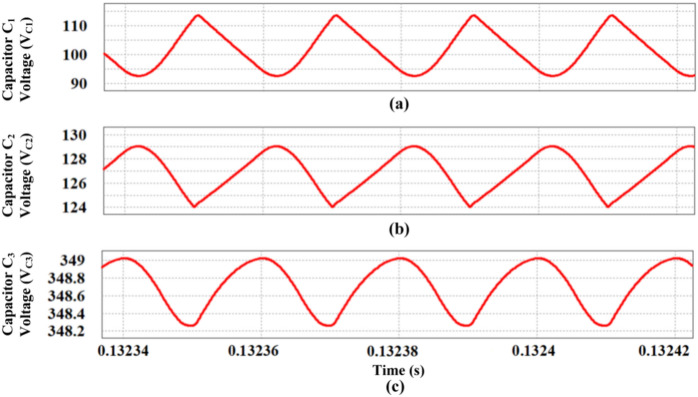
Voltage ripple of capacitors. (**a**) Capacitor C_1_ voltage (V_C1_), (**b**) Capacitor C_2_ voltage (V_C2_), (**c**) Capacitor C_3_ voltage (V_C3_).

The charging and discharging of the capacitors C_1_, C_2_ and C_3_ are observed in Fig. 15 with ripple values of 15, 5 and 3% respectively.

**Fig. 16:**
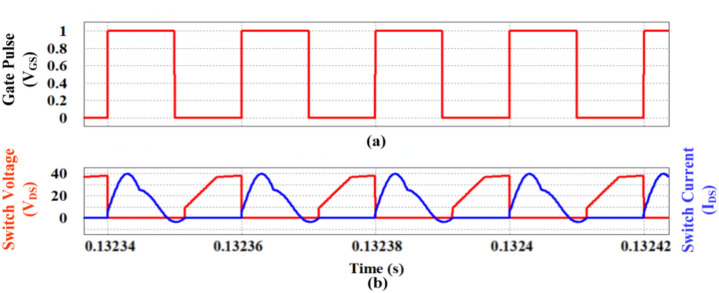
ZCS Simulation response of the proposed converter. (**a**) Gate pulse (V_GS_), (**b**) Controlled switch voltage (V_DS_) and current (I_DS_).

**Fig. 17:**
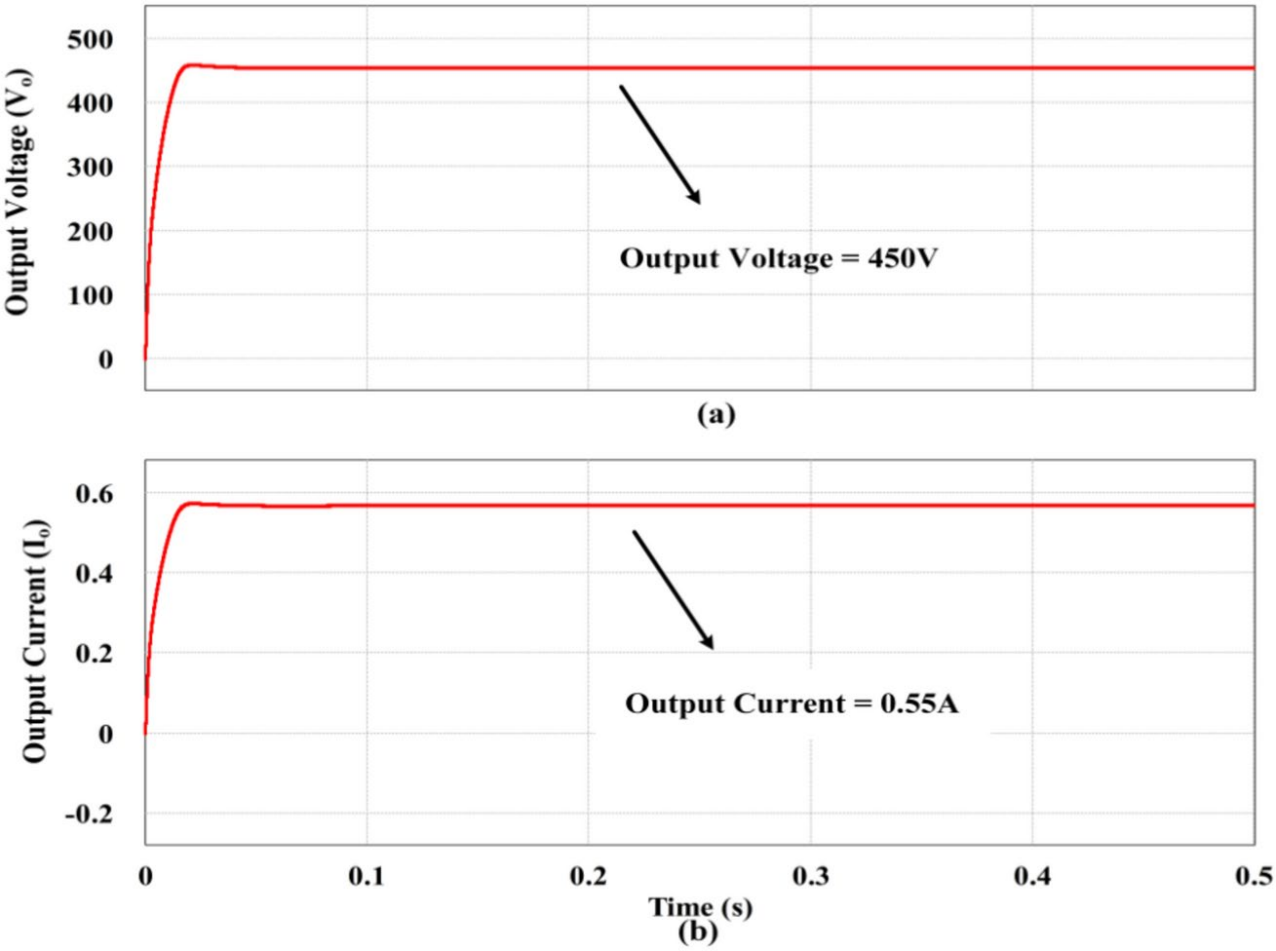
Simulation of Load voltage and current response. (**a**) Output voltage, (**b**) Output current.

The aforementioned simulation waveforms are obtained by operating the controlled switching device (S) under ZCS condition and the same is observed in Fig. 16. It is evident from the Fig. 16a,b that the switch current reaches zero before the falling edge of the gate pulse (V_GS_) and simultaneously the voltage of the switch increases gradually due to the clamping circuit. Consequently, the output voltage of the proposed topology reaches the steady state value of 450 V at 0.55A as portrayed in Fig. 17a,b respectively.

**Fig. 18:**
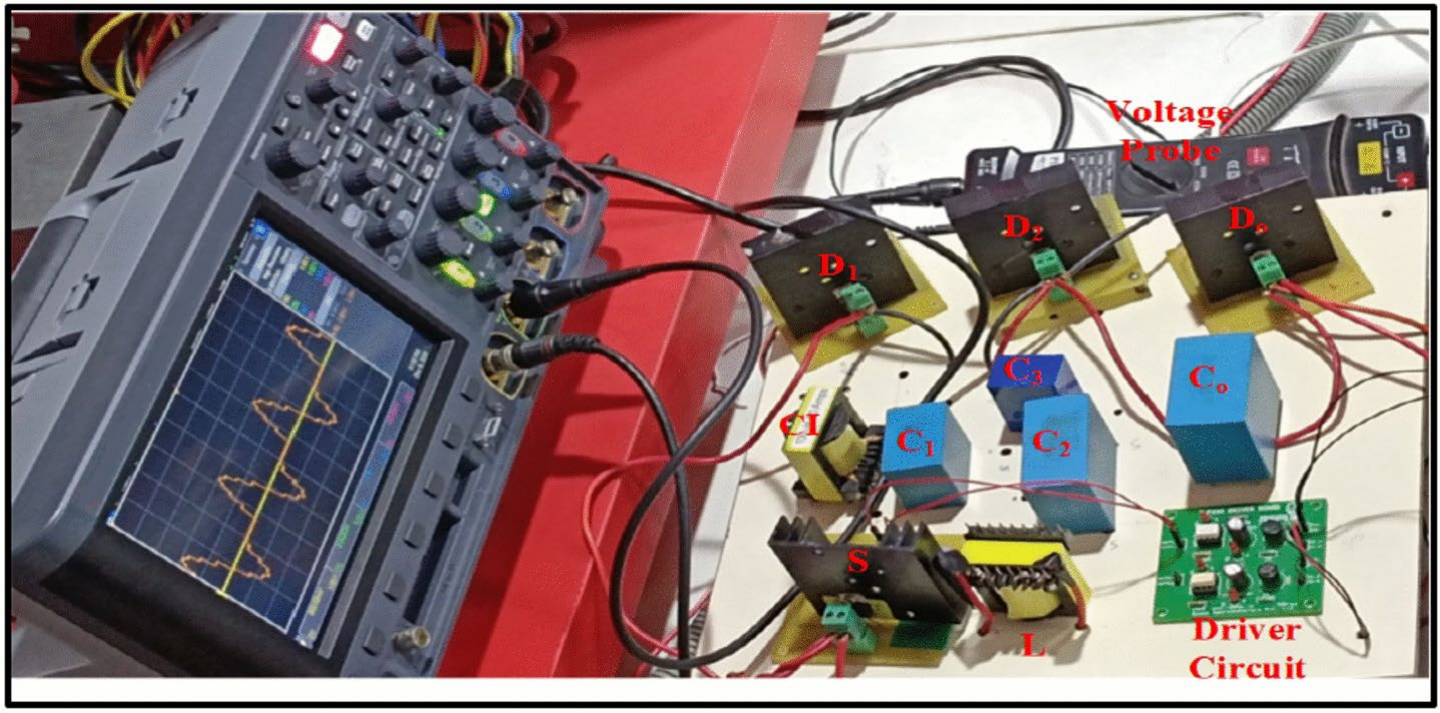
Prototype setup of the proposed converter.

**Fig. 19:**
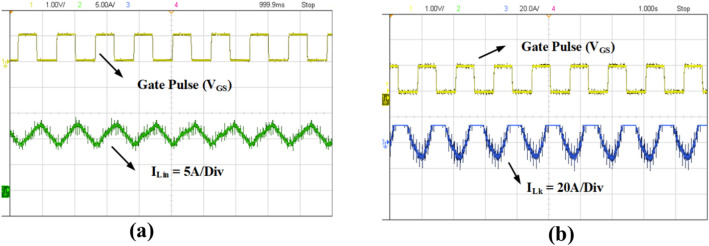
Experimental waveforms of inductor current. (**a**) Input inductor current (I_Lin_), (**b**) Leakage inductance current (I_Lk_).

**Fig. 20:**
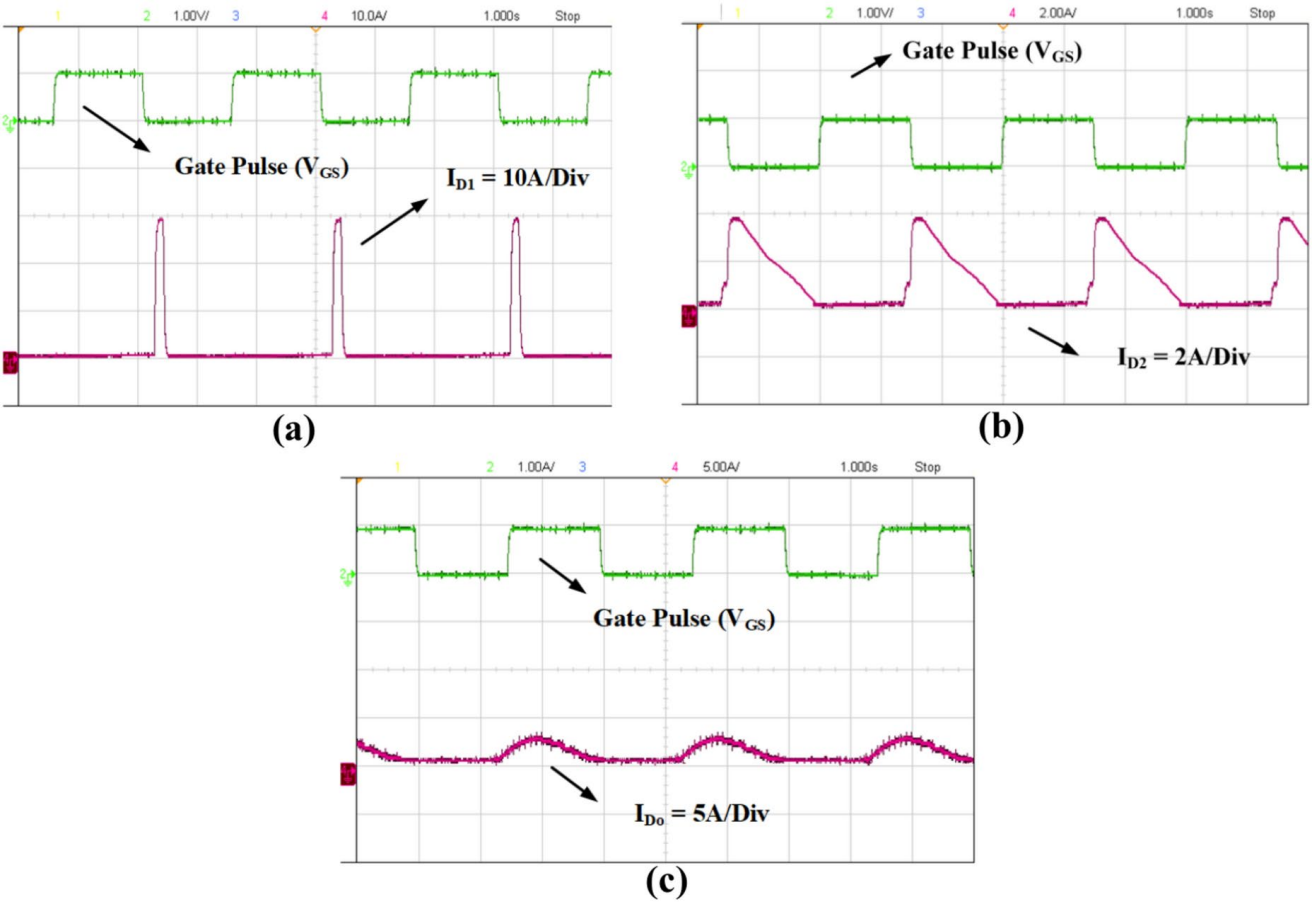
Experimental results of diode currents. (**a**) Diode D_1_ current (I_D1_), (**b**) Diode D_2_ current (I_D2_), (**c**) Diode D_o_ current (I_Do_).

**Fig. 21:**
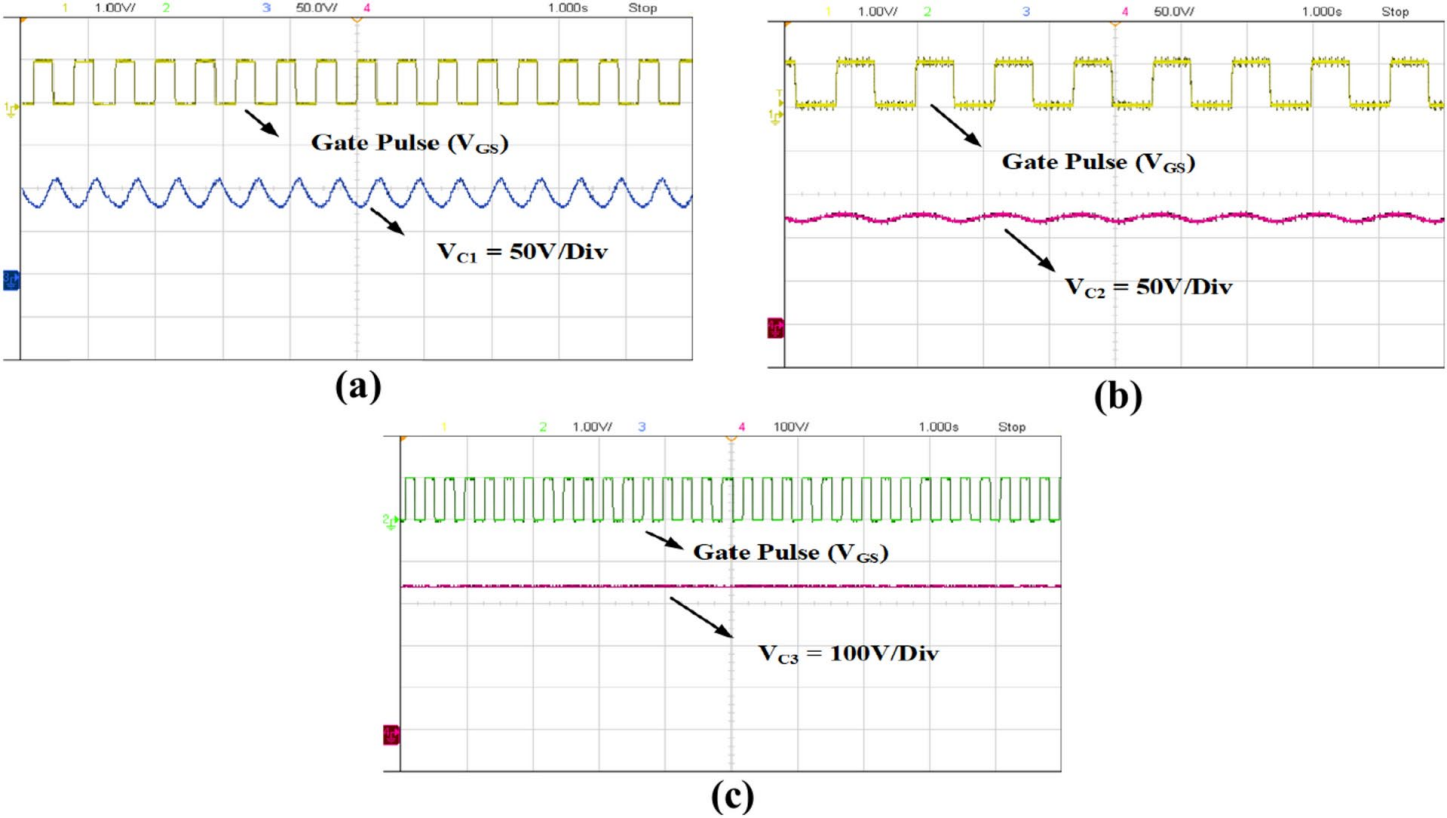
Experimental results: Capacitors voltage ripple. (**a**) Capacitor C_1_ voltage (V_C1_), (**b**) Capacitor C_2_ voltage (V_C2_), (**c**) Capacitor C_3_  voltage (V_C3_).

**Fig. 22:**
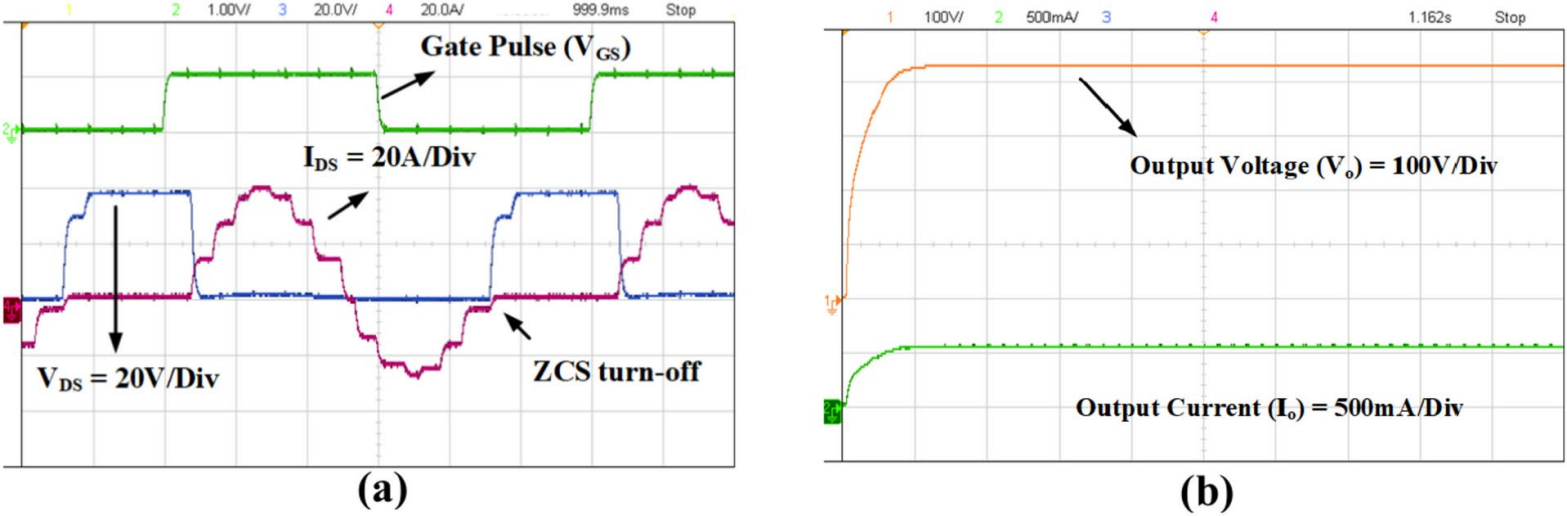
Experimental results: Soft switching (ZCS) of controlled switch (S) and load response. (**a**) Controlled switch voltage and current. (**b**) Load voltage and load current.

The prototype of the proposed high gain converter as shown in the Fig. 18, was constructed using MOSFET IPP076N15N5 and fast acting diodes MUR820. The controlled switch IPP076N15N5 is selected based on low voltage stress of about 40 V as inferred from Fig. 16b and driven by TLP350. The dSPACE controller-MicroLabbox is used to generate the gate pulse for IPP076N15N5 and the experimental results are captured using KEYSIGHT MSOX3014G scope with necessary current probe and voltage differential probe as portrayed in Fig. 18. The experimental results of Figs. 19, 20, 21, 22 are validated with the corresponding simulation results of Figs. 13, 14, 15, 16, 17.

**Fig. 23:**
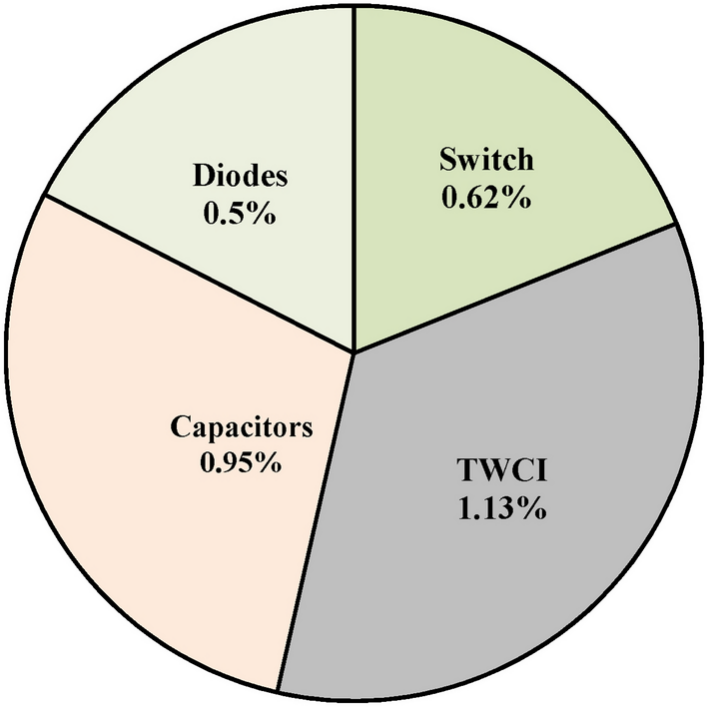
Loss distribution of components in percentage.

The loss calculation^27^ is applied for the proposed converter to obtain the losses of all the components and the corresponding equations are shown in Table 3. Using Table. 3, the efficiency of the proposed converter at 250W is calculated and is equal to 96.5%. The percentage loss distribution is shown in Fig. 23. From Fig. 23 it is observed that the effect of the losses is minimum.

**Table 3:** Power loss calculations of the proposed converter.

Components	Power loss relation	Power loss in watts (D = 0.5)
Switch	${P}_{sw}=\frac{1}{2}{V}_{DS}\left({i}_{DS}^{t=on}\right){f}_{s}+{i}_{DS}^{2}{R}_{{DS}_{on}}$	1.55
Coupled inductor (TWCI)	${P}_{CI(loss)}={i}_{{L}_{in}}^{2}{r}_{{L}_{in}}+{P}_{CI(core)}+{i}_{Lk}^{2}{r}_{eq}$	2.84
Diode (D_1_)	${P}_{D(loss)}={V}_{F}{I}_{D}+{i}_{D}^{2}{r}_{{D}_{on}}$	0.46
Diode (D_2_)		0.46
Diode (D_o_)		0.51
Capacitor (C_1_)	${P}_{c(loss)}={i}_{c}^{2}{ r}_{ESR}$	0.73
Capacitor (C_2_)		0.8
Capacitor (C_3_)		0.75
Capacitor (C_o_)		0.1

*turn-off loss of the MOSFET is neglected due to soft switching action.

**Fig. 24:**
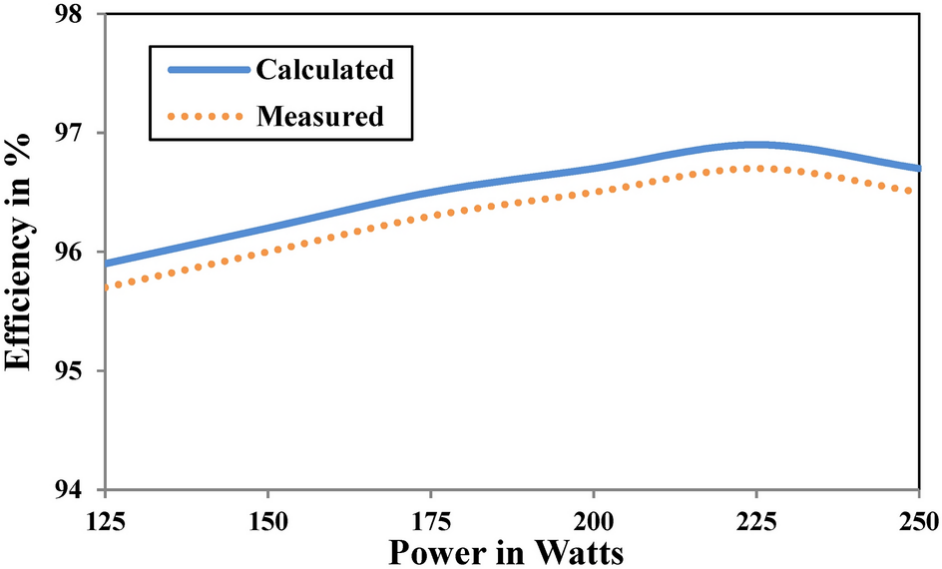
Efficiency of the proposed converter.

The prototype was validated in different power range and at full load an efficiency of 96.5% is achieved and shown in Fig. 24. The results project the feasibility of the proposed topology in the field of electric vehicles.

## Conclusion

This article presents a trans-inverse three-winding CI-based high-gain DC-to-DC converter. The proposed topology achieves high voltage gain by reducing the CI turns ratio and total component count. The clamping circuit performs the ZCS action and lowers the voltage stress during the switch turn-off time. Compared with the other topologies, the proposed topology achieves high voltage gain at a lower turn’s ratio and at a low component count by turning off the main switch using ZCS. The proposed topology is simulated, and the results are validated for the 250W, 450 V prototype under various power conditions using the dSPACE controller. The proposed topology reaches a high efficiency of 96.7% at a power output of 225W.

## Data Availability

The datasets used and/or analysed during the current study are available in the manuscript.
